# Knowledge, attitudes, and interest in orthodontic treatment: a cross-sectional study in adults with stage III-IV periodontitis and secondary malocclusions

**DOI:** 10.1186/s12903-023-03605-8

**Published:** 2023-11-11

**Authors:** Eglė Zasčiurinskienė, Skirgailė Bulotaitė, Krister Bjerklin, Greta Lodienė, Antanas Šidlauskas, Apolinaras Zaborskis

**Affiliations:** 1 Department of Orthodontics, Faculty of Odontology, Medical Academy, Lithuanian University of Health Sciences, Kaunas, LT-44307 Lithuania; 2 Faculty of Odontology, Medical Academy, Lithuanian University of Health Sciences, Kaunas, LT-44307 Lithuania; 3 Department of Orthodontics, Institute for Postgraduate Dental Education, Jönköping, SE-55111 Sweden; 4 Department of Dental and Oral Pathology, Faculty of Odontology, Medical Academy, Lithuanian University of Health Sciences, Kaunas, LT-44307 Lithuania; 5 Faculty of Public Health, Medical Academy, Lithuanian University of Health Sciences, Kaunas, LT-44307 Lithuania

**Keywords:** Periodontitis, Malocclusion, Interest in orthodontic treatment, Adults

## Abstract

**Background:**

An increasing number of patients with advanced stages of periodontitis are seeking for treatment options. The study aimed to determine interest in orthodontic treatment (OT) and its association with oral health status and knowledge about the disease in adult subjects with stage III–IV periodontitis.

**Methods:**

96 subjects ≥ 30 years, with stage III-IV periodontitis agreed to fill in a questionnaire and undergo a comprehensive periodontal-orthodontic examination. The questionnaire included 44 questions: demographic, dental, health related habits, self-perceived overall and oral health, knowledge of periodontitis, and attitude toward OT. The statistical analysis was performed using a paired-sample T-test, Receiver Operating Characteristic (ROC) and binary logistic regression analysis (LRA).

**Results:**

Stage IV periodontitis was observed in 32.3% of subjects and Class II malocclusion was most prevalent (53.1%). More than half of subjects were interested in OT. Oral health, knowledge about periodontitis and age were significant predictors for interest in OT.

**Conclusions:**

Knowledge spread about OT possibilities in advanced stages of periodontitis is very important both for the dental community and older subjects to save natural dentition.

**Supplementary Information:**

The online version contains supplementary material available at 10.1186/s12903-023-03605-8.

## Background

Periodontitis is a chronic polyetiological inflammatory disease caused by numerous factors among which the most important are plaque and aggressive host immune response [1, 2]. The prevalence is up to 50% in the adult population and the severe stages have been reported with the peak of age about 40 years [3–6]. The progression and severity of this chronic polyetiological inflammatory disease are individual for each subject and depend on multiple factors, systemic diseases, and behavioral factors [7, 8]. There is ongoing debate on the influence of malocclusion on periodontitis [9]. Periodontitis is described by stage as per severity and grade by the rate of progression. Severity is based on the number of lost teeth, interdental clinical attachment level (CAL), bone loss, probing depth and secondary occlusal trauma. The disease leads to the attachment loss and gradual loss of teeth, which leads to the decreased posterior occlusal height and affects pathologic tooth migration (PTM), especially in anterior segments [10, 11]. PTM often occurs as an early sign of severe periodontitis and it is a motivation for subjects to seek periodontal and orthodontic treatment [12]. PTM negatively impacts smile aesthetics, leads to impaired function, worsened quality of life, psychosocial well-being and general health [13, 14].

Treatment of advanced stages of periodontitis is usually multidisciplinary, where orthodontic treatment (OT) has an important role in the overall rehabilitation of occlusion [15]. Recent literature revealed that orthodontic treatment does not cause detrimental defects, in some cases results in attachment gain and improvement of alveolar bone levels, however, must be always preceded by periodontal treatment [16–18]. The interest and demand for OT in advanced stages of periodontitis is increasing [12]. Alternatives to orthodontic treatment are splinting or extractions of periodontally affected and migrated teeth, removable and fixed prosthetic appliances on dental implants which have been observed to have lower success rates due to the disease [19–21]. Subjects with periodontitis have been observed to have higher risk for peri-implantitis [22, 23]. Despite the increased demand, OT is rarely included in the overall treatment plan due to a lack of knowledge in the dental community and therefore its possibilities and significance for periodontal subjects are often obscured. Subjects’ knowledge is also of great importance in the timely diagnosis and treatment of the disease [24]. Limited level of knowledge leads to a higher percentage of severe periodontitis and lower interest in treatment [25–27].

Recent literature revealed that more than 50% of subjects with stage III-IV periodontitis needed orthodontic treatment due to the consequences of the disease such as pathologic tooth migration, occlusal trauma and impaired function [11]. However, literature about the subjects’ knowledge and subjects’ willingness to undergo OT in advanced stages of periodontitis is scarce.

Due to the amount of data collected in the present study, the results needed segmented reports. Results on malocclusion prevalence and orthodontic treatment need have been presented in an earlier article [11]. The present study aimed to determine interest in OT and its association with oral health status and knowledge about the disease in subjects with stage III–IV periodontitis.

## Materials and methods

### Ethics and consent to participate

The study was performed according to the Declaration of Helsinki and its later amendments. Ethical approval for the study was granted by the Kaunas Regional Biomedical Research Ethics Committee (protocol No. P1-BE-2-111-2019 approved on March 15, 2021). Written informed consent was obtained from all study subjects after introduction of study purposes, tasks and methods.

### Study design and participants

The study followed a cross-sectional design. A priori required sample size of n = 84 was estimated from the position of the logistic regression analysis using the following parameters: one-tailed, alpha level = 0.05, power = 0.8, and considering effect size (odds ratio) = 1.8 for variables related to interest in OT [28].

Sampling was non-probabilistic using a convenience method. Subjects who were referred to the Department of Dental and Oral Pathology at Lithuanian University of Health Sciences (LUHS), Kaunas, Lithuania (March 2021 to January 2022) were invited to participate in the study asking them to fill in the questionnaire and undergo comprehensive periodontal-orthodontic examination. Eligibility of the subjects was assessed by periodontists during initial periodontal consultation.

To be included in the study, subjects had to have been diagnosed with periodontitis stage III or IV and ≥ 30 years of age. Exclusion criteria were a non-inflammatory periodontal disease, removable prosthetic appliances, multiple missing anterior teeth, pregnant/lactating women, uncontrolled diabetes, and an oncologic diagnosis in the subject’s history.

### Measures

#### Questionnaire

The questionnaire was originally designed for the study combining the questions from other questionnaires found in the literature, which were relevant to the present study [29–32]. Then, the questionnaire validation procedure was performed. The first step in validating was to establish face validity. Professors from the department who played the role of experts evaluated whether the questions effectively captured the topic under investigation, checked the questionnaire for common errors like double-barreled, confusing and leading questions. In the second step, the questionnaire was piloted to test its suitability and relevance for the main survey (n = 35). The pilot testing confirmed content validity of the questionnaire; the variance of answers to the questions met the authors’ expectations. The principal component analysis and assessment of internal consistency were not performed due to the excessive variety of questions making up the scales.

The final questionnaire consisted of 44 questions that involved information about demographic data (3 questions), health related habits (6 questions), self-perceived overall and oral health (17 questions), knowledge of periodontal disease etiology and treatment options (9 questions), and attitude toward orthodontic treatment (9 questions) (Supplement 1). Printed questionnaires were distributed to the subjects before periodontal-orthodontic examination. All questions were given in Lithuanian. Demographic data included gender, age, and education level. Age was dichotomized by < 40 years and ≥ 40 years according to the literature where periodontitis manifestation peak was found at 38 years of age [3]. Education level was assessed as “low” (secondary school/gymnasium or lower) and “high” (high school/university). Smoking was dichotomized according to literature by < 10 cig/day and ≥ 10 cig/day [33].

One of the questions of the questionnaire was “Do you wish to undergo orthodontic treatment?“ (question No. 37). The answers to this question formed a binary variable (Interest in Orthodontic Treatment (OT)) that had two possible outcomes: 0 –“no”, and 1–“yes” (the first outcome combined the respondent’s answers “no”, “don’t know” and missing answers).

The subject’s knowledge of periodontal disease aetiology was assessed by 9 questions (No. 27–35). The answers to each question had a series of options, but only one correct option (marked by * in Supplement 1) that the respondents had to find. Then, the number of correct answers was counted for each respondent (it could vary from 0 to 9; increasing values of the account suggested better knowledge). In analysis, using the Receiver Operating Characteristic (ROC) methodology [34], the sum of correct answers was dichotomized into the binary variable with categories: 0 when “0–5 scores”, and when “6–9 scores”.

Regarding interest in OT, several questionnaire items were also included in the analysis. They considered smoking and alcohol use (questions No. 4–5), systemic diseases (question No. 12), subjective and active assessment of oral health status (questions No. 18–26). Finally, the respondents were asked if they knew about orthodontic treatment options in advanced stages of periodontitis (question No.36). The response was dichotomized into “yes” and “no” (the last category was combined with “don’t know”).

#### Intraoral examination

All intraoral measures were performed by only one calibrated examiner (E.Z.). The evaluation was performed on the six surfaces around each tooth with a periodontal probe (Hu-Friedy PCP-UNC 15, Chicago, IL, USA). The data was recorded in periodontal charts, used for the study.

*Periodontal examination*. The measurements used for periodontal examination complied with classification of periodontitis and may be found in Table 1 [2]. Clinical attachment level (CAL) was chosen as the most important periodontal variable [35]. Measurement analysis included interdental sites that had CAL ≥ 5 mm describing the severity of periodontitis [36]. CAL was also dichotomized into two groups by < 5 mm and ≥ 5 mm [2]. In addition, the percentage of sites CAL ≥ 5 mm within each subject was calculated describing the extent of the disease. By this percentage, subjects were divided into two groups using a 30% cut-off point (≤ 30% and > 30%) [35]. Tooth mobility was assessed by touching the tooth with the index finger on one side and applying a compressive force with an instrument on the other side [37, 38]. Absence of teeth in both dental arches was also recorded and subjects were grouped according to the number of teeth lost (≤ 4/≥5) (the absence of third molars was not considered a loss of teeth) [10].

Stage III or IV of advanced periodontitis was assessed by an experienced periodontist as described in the new classification and case definition (Table 1) [1, 2]. Grading (A, B, C) of periodontitis was adjusted by age, smoking, and diabetes according to the new guidelines for periodontitis case definition (Table 1) [1].

For assessment of occlusal trauma *fremitus* (vibration of the tooth root) was recorded by manual palpation of the labial side of the anterior tooth during clenching to maximum intercuspation [39].

*Orthodontic examination*.

Secondary malocclusion, such as pathologic tooth migration was recorded based on the subject’s complaints about changed tooth positions and clinically by occlusal trauma and spacing/flaring/extrusion of anterior teeth in the maxillary and mandibular dental arch [38]. Orthodontic evaluation included an assessment of sagittal, vertical, and horizontal malocclusion by an experienced orthodontist (E.Z.) (Table 1) The sagittal malocclusion was described using canines because about half of the subjects had lost their first molars. Overjet (OJ) and overbite (OB) was dichotomized into two groups by (≤ 5 mm and > 5 mm) [40–42].

Functional occlusion was assessed by evaluating lower jaw movements during protrusion and laterotrusion. For visualisation of occlusal contacts, 8 μm foil was used. Incorrect guidance in protrusion was registered if only a single incisor or other teeth than incisors guided [41]. The acceptable/correct anterior guidance path was recorded if two, three or all four incisors were in contact during lower jaw movement and all posterior teeth were disoccluded [41]. Lateral movement was considered correct if only canines (canine guidance) or lateral teeth (posterior teeth group function) of the working side were in contact during the function. Incorrect lateral guidance was registered when incisors guided or contacts were present on the non-working side [11].

Orthodontic treatment need was assessed in two ways. The overall orthodontic treatment need including primary malocclusions was assessed by the Dental Health Component (DHC) of the Index of Orthodontic Treatment Need (IOTN) [43]. Very great/great (Grade 5 or 4), borderline (Grade 3), and little/no (Grade 2 or 1) need for orthodontic treatment were registered. Secondarily orthodontic treatment need was judged only by the severity of secondary malocclusion based on occlusal trauma (which could not be treated by alternative methods such as selective grinding), loss of teeth, and severe PTM: flaring and/or extrusion of anterior teeth [11].

To assess the influence of various predictors on the interest in OT following variables were tested: demographic variables (gender, age, education, knowledge, systemic disease) and 20 oral health variables: periodontitis stage and grade, malocclusion primary, occlusal trauma, loss of teeth, spacing/flaring of AT (maxillary/mandibular), extrusion of AT (maxillary/mandibular), periodontal involvement of AT (maxillary/mandibular), extent of periodontitis by percentage of sites with CAL ≥ 5 mm, overbite, overjet, crowding of AT (mandibular/maxillary), mobility of AT (mandibular/maxillary), root disclosure of AT (mandibular/maxillary); also self-reported variables: increased mobility, increased spaces between teeth, satisfaction with the smile aesthetics, stress and orthodontic treatment need (assessed by secondary malocclusions).

**Table 1:** Periodontal and orthodontic intraoral examination variables

Periodontal evaluation	
Periodontal pocket depth (PPD) M, MB, DB, D, DL, ML* sites	Mean value per person
Clinical attachment level (CAL) M, MB, DB, D, DL, ML* sites	Mean value per person; percentage of affected sites
Periodontitis stage	Periodontitis stage III: CAL ≥ 5 mm, PPD ≥ 6 mm, vertical bone loss ≥ 3 mm; tooth loss due to periodontitis of ≤4 teeth Periodontitis stage IV: CAL ≥ 5 mm, PPD ≥ 6 mm, vertical bone loss ≥ 3 mm; tooth loss due to periodontitis of ≥ 5 teeth, secondary occlusal trauma (tooth mobility grade ≥ 2, ≤10 opposing teeth pairs
Periodontitis grade	A- Non smoker, normoglycemic B- Smoker < 10 cig/day or controlled diabetes (blood sugar as low as normal using medication) C- Smoker ≥ 10 cig/day (subjects with uncontrolled diabetes were not included)
**Orthodontic evaluation in maximum intercuspation**	
The sagittal relationship of canines	Class I, Class II, Class III registered by a 2 mm threshold Class II asymmetric (asymmetric canine relationship on both sides)
The sagittal relationship of molars	Was not evaluated due to half of the subjects had lost their first molars
Overbite	Vertical incisor relationship - distance between the mandibular incisor tip and cingulum plateau of the maxillary central incisors in vertical direction
Overjet	Sagittal incisor relationship - distance between the mandibular incisor tip and cingulum plateau of the maxillary central incisors in vertical direction
Crossbite	Anterior or/and posterior crossbite was registered

Notes. M- mesial, MB- mesiobuccal, DB- distobuccal, D- distal, DL- distolingual, ML- mesiolingual, OT- orthodontic treatment

#### Reliability of measurements

The clinical attachment level (CAL) was selected for reliability measurements as recommended in the literature [44]. The intraclass correlation coefficient (ICC) yielded a value of 0.93 (95% CI: 0.87, 0.95; p < 0.001), and interclass agreement between examiners was 0.95 (95% CI: 0.92, 0.96; p < 0.001). Periodontist, who performed assessments of periodontal diagnosis, was calibrated with two other experienced periodontists for assessment of stage and grade. Inter-examiner reliability yielded Cohen’s kappa coefficient of value 0.92 and intra-examiner reliability of 0.97. Calibration for the assessment of PTM of principal investigator (E.Z.) was performed by two experienced orthodontists, which resulted in the values of Cohen’s kappa coefficient of 0.81 (for evaluation in maxillary AT) and 0.87 (for evaluation in mandibular AT). Any disagreement between the examiners was solved by thorough discussion [11].

### Statistical analysis

The data were analyzed using SPSS Statistics version 27.0 for Windows (IBM Corp., Armonk, NY, USA). The analysis was carried out in stages. First, frequency analysis and descriptive statistical analysis were conducted to examine the characteristics of the variables. The significance of the difference in the variable prevalence across groups was evaluated using the z-test or the chi-squared test when there were two or more than two groups respectively. Next, to address the research questions, three models were analyzed through binary logistic regression analysis (LRA). Model 1 attempted to verify the effect of separate oral health variables on the subject’s interest in orthodontic treatment (OT) using univariate LRA. Model 2 attempted to examine the effect of the subject’s knowledge regarding periodontitis on interest in OT adjusting LRA by demographic variables. Finally, in Model 3, the common effect on subjects’ interest in OT of all significant predictors that were identified in Model 1 was examined using multivariate LRA with the Forward LR method of entering variables. The regression models were also tested for multicollinearity, but any multicollinearity problems were diagnosed (for all variables the statistic VIF was less than 10; the highest value (3.78) of this statistic had periodontal stage). Results were reported as odds ratios (OR) with *p*-values. In all statistical tests, significance was considered when *p* < 0.05 and high significance when *p* < 0.01.

## Results

### Sample characteristics

**Fig. 1:**
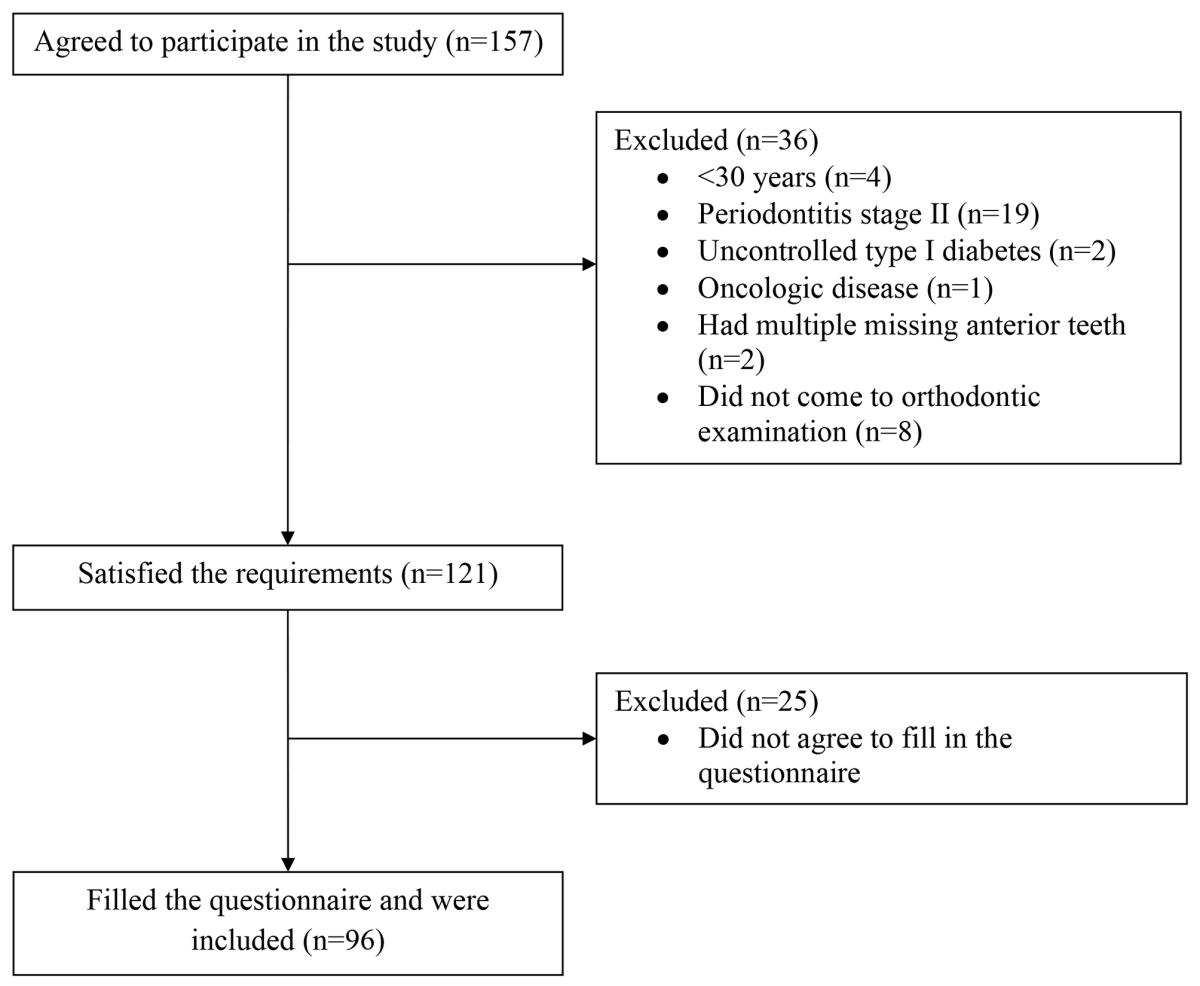
Flowchart of present study

Flowchart of the study participants is shown in Fig. 1. As can be seen from the presented chart, out of the relevant 121 subjects, only 96 (79.3%) subjects entered the analysed sample as they agreed to fill in the questionnaire. Of them, 29 (30.2%) were males and 67 (69.8%) were females. The mean age of the subjects was 45.7 (SD 10.2) years (range 30–78 years). 60.4% of subjects were > 40 years of age. Most (69.8%) of enrolled subjects had a high school or university education. 10% were heavy smokers (≥ 10cig/day) and 72.3% used alcoholic drinks at least monthly. The systemic disease was found in 42.7% of enrolled subjects, and it was more prevalent among males (62.1%, p = 0.012) as well as in the age group > 40 years than in the younger age group (55.2%, p = 0.002). The most common systemic disease was hypertension; it accounted for 51.2% of the structure of listed diseases.

### Periodontal and orthodontic assessment of the sample

Results from the periodontal and orthodontic examinations of subjects are presented in Table 2. It can be seen that stage IV periodontitis, was observed in about one-third (32.3%) of the subjects (p < 0.001). Grade C was more prevalent (77.4%) among stage IV periodontitis compared with stage III (26.2%) periodontitis subjects (p < 0.001). Interest in orthodontic treatment (OT) was more observed among subjects with stage IV (p < 0.001).

The mean clinical attachment level (CAL) was 3.42 mm (95% CI: 3.19; 3.65). The majority (89.6%) of subjects had CAL ≥ 5 mm at least in one of the anterior teeth (AT), affecting more maxillary than mandibular AT (p = 0.08) (Table 2). At least one pair of AT with occlusal trauma was identified in 84.4% of subjects. More than half (53.1%) of the subjects had Class II malocclusion. However, a large part (39.2%) had asymmetric class II (Class II on one side and Class I on the other). The main and most prevalent secondary malocclusion was spacing of AT found in 60.4% of subjects, which was more prevalent in maxillary than in mandibular AT, (p < 0.001), and extrusion of AT found in 62.5% of subjects, which was similarly prevalent in AT of both dental arches (p = 0.78). Crowding was more prevalent in the mandibular than in the maxillary dental arch (57.3% vs. 32.3%, p < 0.001). Males had significantly higher rates of several periodontal and orthodontic impairments than females, respectively extrusion of AT (82.8% vs. 53.7%, p = 0.01),percentage (≤ 30%/>30%) of sites with CAL ≥ 5 mm (52.2% vs. 19.4%, p < 0.001), maxillary AT with CAL ≥ 5 mm (89.7% vs. 68.7%, p = 0.03), mandibular AT with CAL ≥ 5 mm (79.3% vs. 56.7%, p = 0.04). Some impairments were more common in the younger (≤ 40 years) than older (> 40 years) age group, respectively, crowding of upper teeth (44.7% vs. 25.0%, p = 0.046), crowding of lower teeth (76.3% vs. 44.6%, p = 0.002), but the later one was significant only in the female group.

Assessment by DHC-IOTN yielded that 7.3% had very great (Grade 5), 46.9% great (Grade 4), and 19.8% borderline OT need. When judged by secondary malocclusions 59.4% subjects were assessed to have OT need due to PTM and/or occlusal trauma and/or incorrect functional guidance; of them 87.1% subjects were with of stage IV and 46.2% of stage III periodontitis (p = 0.001).

**Table 2:** Descriptive characteristics of periodontal and orthodontic intraoral evaluation in the studied sample (n = 96)

Periodontal and orthodontic intraoral evaluation	n	(%)
Stage of periodontitis:		
III	65	(67.7)
IV	31	(32.3)
Periodontitis grade:		
A	35	(36.5)
B	20	(20.8)
C	41	(42.7)
Malocclusion primary:		
no	9	(9.4)
Class I	30	(31.3)
Class II-1	22	(22.9)
Class II-2	9	(9.4)
Class II asymmetric class	20	(20.8)
Class III	6	(6.3)
AT with occlusal trauma:		
yes	81	(84.4)
no	15	(15.6)
Loss of teeth:		
none	24	(25.0)
1–4 teeth	54	(56.3)
5 or more teeth	18	(18.8)
Secondary malocclusion:		
a) spacing/flaring maxillary:		
yes	51	(53.1)
no	45	(46.9)
b) spacing/flaring mandibular:		
yes	27	(28.1)
no	69	(71.9)
c) extrusion AT maxillary:		
yes	35	(36.5)
no	61	(63.5)
d) extrusion AT mandibular:		
yes	37	(38.5)
no	59	(61.5)
Lost posterior support of occlusion:		
yes	49	(51.0)
no	47	(49.0)
Percentage of sites with CAL≥5 mm:		
≤30%	29	(30.2)
> 30%	67	(69.8)
Maxillary AT with CAL≥5 mm:		
yes	72	(75.0)
no	24	(25.0)
Mandibular AT with CAL≥5 mm:		
yes	61	(63.5)
no	35	(36.5)
Overbite:		
< 5 mm	70	(72.9)
≥ 5 mm	26	(27.1)
Overjet:		
< 5 mm	70	(72.9)
≥ 5 mm	26	(27.1)
Crossbite:		
No crossbite	59	(61.5)
posterior	28	(29.2)
anterior	7	(7.3)
scissors bite	1	(1.0)
crossbite all	1	(1.0)
Crowding of upper teeth:		
yes	31	(32.3)
no	65	(67.0)
Crowding of lower teeth:		
yes	55	(57.3)
no	41	(42.7)
Mobility of AT:		
yes	64	(66.7)
no	32	(33.3)
Orthodontic treatment need:		
yes	57	(59.4)
no	39	(40.6)

AT: anterior teeth; CAL: clinical attachment level

### Subjective assessment of oral health

The distribution of subjects’ responses to questions about their oral health related issues is shown in Table 3. The survey yielded that about half (52.1%) of the subjects had periodontal treatment before, while only 15.6% of the subjects have had orthodontic treatment. A large number of subjects complained of serious oral health problems: bleeding of the gums (65.6%), mobility of teeth (64.6%), increased spaces between teeth (71.9%). Undoubtedly, these reasons could have caused stress or dissatisfaction with the smile aesthetics, and as can be seen from the data, there were quite many such cases.

**Table 3:** Subjective assessment of oral health in the studied sample (n = 96)

Subjects’ response	n	(%)
Had periodontal treatment before:		
yes	50	(52.1)
no	44	(45.8)
missing data	2	(2.1)
Had orthodontic treatment before:		
yes	15	(15.6)
no	79	(82.3)
missing data	2	(2.1)
Noticed gums bleeding:		
yes	63	(65.6)
no	33	(33.4)
Noticed mobility of teeth:		
yes	62	(64.6)
no	22	(22.9)
lost teeth because of increased mobility	12	(12.5)
Noticed increased spaces between teeth:		
yes	69	(71.9)
no	27	(28.1)
Satisfied with the smile aesthetic:		
never been satisfied	25	(26.1)
not satisfied in the recent days	39	(40.6)
yes	32	(33.3)
Was stressed:		
a lot	18	(18.8)
sometimes	51	(53.1)
no	27	(28.1)

### Interest in orthodontic treatment

The distribution of subjects’ responses to questions about their interest in orthodontic treatment (OT) is shown in Table 4.

It is seen that over half (56.3%) of the subjects expressed an interest in OT. Others were not interested in this treatment or were undecided (in further analysis, these groups of subjects were combined into one, which was considered as the group of subjects not interested in orthodontic treatment). The responses were almost equally distributed between the genders (51.7% of the men, and 58.2% of the women, *p* = 0.556). The proportion of subjects who expressed interest in OT was significantly higher in the younger (≤ 40 years) age group (73.7% vs. 44.8%, *p* = 0.005). Interest in orthodontic treatment was more often observed among subjects with stage IV (*p* = 0.014) and grade C periodontitis (*p* = 0.007).

In addition, it was found that those who reported interest in OT had the following motives to undergo orthodontic treatment: maintaining their own teeth – 29.6%, improve aesthetic appearance and function – 16.7%, and both above motives – 53.7%. However, many of them were concerned about the price of the treatment (41.4%). The length of the treatment was not the main concern (17.8%). Although most of the interested subjects (90.7%) would agree to pay for the treatment, two-thirds of them were concerned about the price. 44.4% agreed to get treatment with braces, and 35.2% with clear aligners. From those who responded negatively or did not know, 76.2% of subjects were older than 40 years.

**Table 4:** Descriptive characteristics of interest in orthodontic treatment in the studied sample (n = 96)

Subjects’ response	n	(%)	p
Interest in orthodontic treatment:			
yes	54	(56.3)	
no	19	(19.7)	
don’t know	23	(24.0)	
Frequency of “yes”, by			
Gender:			
males (n = 29)	15	(51.7)	0.556
females (n = 67)	39	(58.2)	
Age:			
≤ 40 years (n = 38)	28	(73.7)	0.005
> 40 years (n = 58)	26	(44.8)	
Education:			
≥high school	38	(58.5)	0.451
≤gymnasium	14	(50.0)	
Stage of periodontitis:			
III	31	(47.7)	0.014
IV	23	(74.2)	
Periodontitis grade:			
A	13	(37.1)	
B	11	(55.0)	0.007
C	30	(73.2)	

### Subjects’ knowledge of periodontal disease and orthodontic treatment

Subjects were asked to assess their knowledge of periodontal disease aetiology and treatment. Looking through their responses (Table 5), low knowledge about systemic diseases and pregnancy’s influence on periodontitis can be noticed; there was only 53.1% and 26.0% of respondents provided correct answers to the relevant questions (Table 5).

The sum of correct answers varied from 0 to 9 with a mean of 6.45 (SD 1.65) and a median of 7. Regarding the classification of the subjects by their interest in orthodontic treatment (OT), the ROC analysis found an optimal decision threshold of 5.5 scores to divide subjects into groups with “low” and “high” knowledge. Thus, the first group of respondents who answered no more than 5 questions correctly, included 21.9% (n = 21) of subjects, while the second group of respondents who answered 6 or more questions correctly, included 78.1% (n = 75) of subjects. Such division ensured a sensitivity of 0.87 and a specificity of 0.67. A higher but not significant level of knowledge was observed among women (82.1%), younger age (81.6%), and higher education groups (79.1%). No significant dependence of their knowledge on gender, age, and education was found (p > 0.05).

**Table 5:** Percentage of correct answers to test questions about periodontitis in studied subjects, by their interest in orthodontic treatment (n = 96)

Question	All subjects (n = 96)		Subjects interested in OT (n = 54)		Subjects not interested in OT (n = 42)		p
	n	(%)	n	(%)	n	(%)	
How is a doctor who specializes in treatment of periodontal tissue diseases called?	69	(71.9)	40	(74.1)	29	(69.0)	0.587
What is the primary cause of periodontal diseases?	71	(74.0)	43	(79.6)	28	(66.7)	0.151
How often is it recommended to get for professional oral hygiene?	66	(68.8)	39	(72.2)	27	(64.3)	0.405
Does systemic diseases have an influence on periodontal tissues?	51	(53.1)	31	(57.4)	20	(47.6)	0.340
What it is the effect of smoking for periodontal tissues?	77	(80.2)	49	(90.7)	28	(66.7)	0.003
What symptoms show the start of gingival inflammation (gingivitis)?	87	(90.6)	52	(96.3)	35	(83.3)	0.031
Is it possible to lose a tooth because of progressive not treated periodontal disease?	93	(96.9)	54	(100.0)	39	(92.9)	0.046
Does pregnancy have an influence on periodontal diseases?	25	(26.0)	12	(22.2)	13	(31.0)	0.334
Which methods are used for the treatment of periodontal diseases?	80	(83.3)	45	(83.3)	35	(83.3)	1.000

Note: OT - orthodontic treatment

### Relationship between interest in OT, knowledge and oral health status

Primarily, we calculated the odds ratio (OR) as a measure to assess the strength of the association between subjects’ interest in orthodontic treatment (OT) and each variable. The results of this analysis are shown in column Model 1 of Table 6. It can be seen that the likelihood of subjects’ interest in OT was significantly lower in older age (> 40 years) and the presence of systemic diseases but was independent of gender and education level. Subjects were more likely to have interest in OT due to more pronounced symptoms of periodontitis and malocclusion, whereas this association was significant for 6 (out of all) tested variables. Interestingly, there was a significant association between the clinical judgment of the need for OT and self-interest in OT (OR = 2.9, p = 0.01). The subjects whose knowledge of periodontitis was highly rated had 3.4 times higher odds (*p* = 0.02) than subjects with poorer knowledge. A significant relationship between subjects’ interest in OT and their knowledge (OR = 3.3, p = 0.03) was also confirmed when the analysis was performed by adjusting data for demographic variables (column Model 2 of Table 6).

Finally, the association between subjects’ interest in OT and their knowledge was assessed by adjusting data for both demographic and clinical variables (column Model 3 of Table 6). The LRA with the forward LR option reaffirmed once again that subjects’ knowledge in periodontitis is an important factor related to the subjects’ interest in OT (OR = 5.91, p = 0.02). In addition to this factor, the interest in OT in subjects was dependent on several clinical factors, the most important of which can be seen in Table 6. In this step of the analysis (Model 3), it is interesting to note that subjects’ interest in OT was no longer related to their age, but became related to gender, whereas females, compared to males, were 5.9 times more likely to be interested in OT (p = 0.02).

**Table 6:** Effect of demographic variables, knowledge, periodontal and orthodontic health status on interest in orthodontic treatment, by different models of variable selection

Variable (compared categories)	Model 1		Model 2		Model 3	
	OR	*p*	OR	*p*	OR	*p*
Gender:						
females vs. males	1.30	0.557	0.94	0.899	5.93	0.015
Age:						
≤40 years vs. > 40 years	3.45	0.006	3.57	0.008	0.62	0.203
Education:						
≥high school vs. ≤ gymnasium	1.41	0.452	1.14	0.793	0.45	0.504
Knowledge:						
≥6 scores vs. ≤ 5 scores	3.36	0.020	3.27	0.032	5.91	0.015
Systemic disease:						
no vs. yes	2.44	0.037				
Periodontitis stage:						
IV stage vs. III stage	3.15	0.017			6.04	0.014
Periodontitis grade:						
B vs. A	2.07	0.202				
C vs. A	4.62	0.002				
Extrusion of AT ^a^:						
yes vs. no	5.75	< 0.001			4.51	0.023
Mandibular AT with CAL ≥ 5 mm:						
(yes vs. no)	4.24	0.001			3.77	0.049
Crowding of lower teeth:						
yes vs. no	4.33	0.001			7.82	0.005
Orthodontic treatment need:						
yes vs. no	2.88	0.014				
Noticed increased mobility of teeth:						
yes vs. no	3.73	0.011			2.61	0.106
Satisfied with the smile esthetic:						
no vs. yes	2.61	0.031				

Notes. Of the oral health status variables, only those with a significant association with IOT are included in the table; significant values (*p* < 0.05) are in bold; ^a^ extrusion of maxillary and mandibular anterior teeth was combined into one variable due to insignificant difference in their rates. AT: anterior teeth; CAL: clinical attachment level; OR: odds ratio

## Discussion

Orthodontic treatment (OT) is more often considered as part of the overall treatment and occlusal rehabilitation of subjects with advanced stages of periodontitis [15]. Orthodontic movement of periodontitis affected and migrated teeth is gaining popularity due to the effects of improved periodontium, possibility of saving natural teeth and also due to the literature that shows higher risk for the development of peri-implantitis in subjects with periodontitis [16, 45]. OT need in subjects with stage III-IV periodontitis was discussed in recent publication [11]. However, we found the importance in assessing the interest in OT in this special group of subjects. The present study focused on analyzing the interest in orthodontic treatment (OT) among individuals with stage III and IV periodontitis. Additionally, it aimed to examine how this interest in OT is related to their oral health status and their knowledge about the disease. The sample analysis showed that half of the enrolled subjects were older than 40 years, which is probably related to the fact that periodontitis prevalence increases with age [5]. Nearly half of the subjects had systemic diseases, which is also related to older age and advanced stages of the disease [46]. The consistency of findings between the present study and a recent study conducted in Germany, where more than half (68%) of subjects with stage III-IV periodontitis expressed interest in orthodontic treatment (OT) [29]. This similarity in results suggests that the interest in OT among individuals with advanced periodontitis may be a trend that extends beyond a single study or location [12]. Interestingly, the aforementioned study found that many of the periodontally affected subjects were never offered orthodontic correction [12].

Interest in OT in the present study was affected by a subjective factor, such as compromised smile esthetics, which was observed in 2/3 of the included subjects and which is a well described general factor in many subjects seeking OT [12, 47]. Improved smile esthetics has been found to impact self-esteem and self-confidence and improve psychological well-being [48–50]. Most of those who were interested in OT agreed to get treatment with braces or clear aligners. Another factor, which affected interest in OT was willingness to maintain natural teeth, which was also found in a study mentioned above as encouraging motive to undergo OT [12]. Among factors, affecting interest in OT in the present study, was younger age (≤ 40 years) and systemic health. In contrast, the study, performed in Germany, observed a higher trend towards interest in OT with increasing age due to the willingness to save natural teeth [12]. According to the study performed in Korea, respondents aged over 40 considered themselves “too old” for OT [47]. Unfortunately, high OT price was also observed to be one of the main issues when seeking treatment [47]. The price concern was also found in the present study.

Univariate analysis also revealed that significant motives for subjects to express interest in OT were objective periodontal (e.g., stage and grade of periodontitis) and secondary orthodontic changes (e.g., extrusion of AT). Self-perceived mobility of teeth, which is often associated with self-perceived risk for tooth loss, was also found to be an important factor (Table 5). According to the literature, only periodontitis stage IV is associated with need of OT as a part of complex rehabilitation of the occlusion [2]. However, when clinically judged in the present study, OT need due to secondary malocclusions was assessed in 87.1% subjects with stage IV and as many as 46.2% subjects with stage III periodontitis. In our earlier article we described OT need in this particular group of subjects and found that it depended not only on periodontitis stage or grade, but also on primary (e.g., crowding, Angle II or III) and/or secondary malocclusions (e.g., impaired functional guidance or increased spacing) [11]. Interestingly, class III malocclusion had a high odds ratio for PTM, such as spacing and/or flaring, especially in subjects with tongue habit [11]. So, it is important to underline, that not only subjects with stage IV periodontitis with need of complex rehabilitation, but also subjects with stage III periodontitis need orthodontic corrections. This was judged due to primary and/or secondary malocclusions, which, if not corrected at this stage, may continue to worsen, leading to tooth loss and with time development to more advanced secondary malocclusions, which are classified as stage IV periodontitis. Heavy occlusal contacts induce risk for further periodontal breakdown and migration of teeth, especially in cases of untreated periodontitis [51]. The literature has also described that teeth, exposed to traumatic occlusal interferences, have worse healing after periodontal therapy [52].

Also, in the present study, we found a significant association between the clinical judgment of the OT need and self-interest in OT. However, as mentioned above, stage of periodontitis does not reflect OT need and cannot be the only criteria used for assessing subjects’ interest in OT. Due to scarcity of the literature in this field, we were not able to compare our results.

The list of significant factors was supplemented by subjects’ knowledge about periodontitis and OT. In our questionnaire survey, subjects answered questions to test their knowledge about periodontitis aetiology and OT. The relatively high rates of correct responses regarding the causes of periodontitis in the present study are encouraging findings and similar to those obtained from a survey of Poland’s population (74% vs. 81% and 80% vs. 85%, respectively) [27]. The finding that the rates of correct answers to questions about the influence of systemic diseases and pregnancy on periodontitis were relatively low is noteworthy. In total, we considered that 78% of surveyed subjects had “high” knowledge in the interested area. However, it’s noteworthy that knowledge deficits were more visible among older individuals (those older than 40 years) and subjects with lower education levels [27, 53]. Based on the findings of the present study, the information from dental care providers is crucial for subjects with periodontitis to obtain reliable knowledge about possibilities of saving natural teeth [16, 17]. Furthermore, literature suggests, that personalized strategy is important for successful multidisciplinary treatment of the disease [54].

The results of the present study showed that subjects’ interest in OT is associated with subjects’ knowledge regarding periodontal disease which was never described in the earlier literature [54]. It is also important to recognize genders’ significance, as women were found to be more interested in OT than men (Table 5). This could be explained by the fact that some clinical symptoms (e.g., periodontal involvement and extrusion of AT) depended more significantly on gender than on age.

Highlighting the significance of OT in the aging population is important due to its unique possibility of saving teeth [16, 17].

### Limitations

One of the limitations of the present study relatively small sample size, presumably due to the reluctance of the subjects to participate. From the original sample that matched the inclusion criteria for the study, 79.3% of subjects agreed to fill in the questionnaire. The fact that the study was conducted during the COVID-19 pandemic and that many relevant subjects were lost due to pandemic-related factors is an important contextual detail and limitation to consider. Another factor is that we included only subjects with stage III-IV periodontitis which is about 10% of the population [3]. However, the number of subjects enrolled in the survey was sufficient to obtain a high level of significance, for example, in testing associations. The limitation that the study group consisted of subjects seeking professional periodontal help at the university dental clinic is an important consideration when interpreting the study’s findings and generalizing the results and cannot be compared to the entire Lithuanian population affected by severe periodontitis. Also, it is worth mentioning that one quarter (24%) of the subjects were undecided to express their interest in OT, but in analysis, this group was combined with the group of subjects who reported no interest in OT. Such an approach could overestimate the significance of the statistical conclusions. However, recalculations without “undecided” subjects did not show any significant changes in the findings. A cross-sectional design of the study limits validity of its findings, as only associations between variables but not causation between them could be suggested [55]; longitudinal studies are needed to test the predictive values of studied variables on the subjects’ decisions. According to the health behavior models, other aspects, for instance, subjects’ health-seeking behavior, is equally important in motivating subjects’ interest in a healthy lifestyle as well as, if necessary, in treatment options [56].

## Conclusions

More than half of the subjects were interested in orthodontic treatment (OT). Subjects with periodontitis stage IV, grade C, absence of systemic disease, and younger than 40 years were more interested in OT. Oral health variables: periodontitis, extrusion, self-reported mobility, and crowding of anterior teeth were significant predictors for interest in OT. Interest in OT was also significantly associated with subjects’ knowledge about periodontitis. Consequently, subjects who were > 40 years old had lower knowledge and lower interest in OT.

Spread of the knowledge about orthodontic treatment possibilities is important both for the dental community and patients, especially in advanced stages of periodontitis to save the natural dentition.

### Electronic supplementary material

Below is the link to the electronic supplementary material.

Supplementary Material 1

## Data Availability

The data is available from the corresponding author, upon reasonable request.

## Abbreviations

OT: Orthodontic treatment
LRA: Logistic regression analysis
AT: Anterior teeth
CAL: Clinical attachment level
PTM: Pathologic tooth migration
PPD: Periodontal pocket depth
MB: Mesiobuccal
B: Buccal
DB: Distobuccal
ML: Mesiolingual
L: Lingual
DL: Distolingual
OJ: Overjet
OB: Overbite
DHC: Dental Health Component
IOTN: Index of Orthodontic Treatment Need
