# Supplementary material for: Knowledge, attitudes, and interest in orthodontic treatment: a cross-sectional study in adults with stage III-IV periodontitis and secondary malocclusions
Source: BMC Oral Health. 2023 Nov 11;23:853. doi: 10.1186/s12903-023-03605-8 (PMC10640755; doi:10.1186/s12903-023-03605-8)
Supplement: Supplementary file 1 — Supplementary Material 1 [file 12903_2023_3605_MOESM1_ESM.docx]

**QUESTIONNAIRE**

**Part one – general questions and complaints**Circle one of the answers, write numbers or text

| **No.** | **Question** | **Answer options** | | | | |
| --- | --- | --- | --- | --- | --- | --- |
| 1. | Sex | Male | Female |  | | |
| 2. | Age | ………………… years | |  |  |  |
| 3. | Education | Primary | Basic (8–10 years) | Secondary or gymnasium (12 years) | Higher school | University |
| 4. | Do you smoke? | No | Sometimes | Less than 10  cigarettes/day | 10–20 cigarettes/day | More than 20 cigarettes/day |
| 5. | What is your alcohol consumption? | No | Once or more per month | Once or more per week |  |  |
| 6. | When was your last dental appointment? | <6 months ago | 6-12 months ago | >1 year ago |  |  |
| 7. | How often do you brush your teeth? | Once per day or less often | Twice or more per day |  |  |  |
| 8. | Do you use dental floss? | Everyday | Sometimes | No, I do not |  |  |
| 9. | Do you use interdental brush? | Everyday | Sometimes | No, I do not |  |  |
| 10. | How would you describe your diet? | Eating regularly, balanced food | Eating not regularly, imbalanced food | Trying to eat wholesome food, but not managing to succeed every time | |  |
| 10.A | How often do you eat sweets? | Yes, everyday | Once or more per week | Rarely | No |  |
| 10.B | Are you overweight? | Yes | No |  | | |
| 11. | Are you wearing removable dentures? | Yes | No |  | | |
| 12. | Do you have any systemic diseases? | Diabetes | Heart diseases or hypertension | Blood diseases | Autoimmune diseases | No  Other.................... |
| 13. | Does anyone in your family have any periodontal diseases history? | Yes  If “Yes”, then who ........................................................... | | No |  | |
| 14. | Do your gums bleed? | Yes, when brushing teeth | Yes, when eating hard food | I can taste blood often | No |  |
| 15. | Have you had any periodontal treatment before? | No | Yes, less than 2 years ago | 2 or more years ago | Treatment was start, but not finished | Other information .............................. |
| 16. | Have you been diagnosed with an incorrect bite in the past? | Yes  If “Yes”, then when and what kind ................................................................... | | No |  |  |
| 17. | Have you had any orthodontic treatment before? | Yes  If “Yes”, then when?)  …………………….. | | No |  |  |
| 18. | Do you have teeth with root disclosure or gum recession? | Yes | No |  | |  |
| 19. | Do you have any teeth with increased mobility? | No | Yes, I have one or more teeth, with increased mobility | | I have lost teeth, because of their increased mobility | |
| 20. | Have you noticed any changes in bite/ teeth, that migrated, after molar extraction? | Yes, I did notice teeth place changes after extraction | | No, I did not | I have all molars |  |
| 21. | Have you been advised to get orthodontic treatment in the past? | No | Yes, but treatment has not been started (why?) Write below ……………………………………… | | Yes, orthodontic treatment was started, because of teeth migration, before........ years. | |
| 22. | Do you consistently feel bad breath in mouth? | Yes | No |  | |  |
| 23. | Have you noticed increased spaces between teeth? | Yes | No |  | |  |
| 24. | Do you feel pain or discomfort in your gingiva after eating? | Yes, I feel it every time | Sometimes, when eating hard food | | No, I do not |  |
| 25. | Do you have any complaints about your poor smile esthetic? | No | Yes, the smile esthetic has gone poor during the recent days | | I have never been satisfied with my smile esthetic | |
| 26. | Are you stressed? | Yes, I feel a lot of stress | | Sometimes | No, I do not |  |

**Part two – knowledge and attitude towards orthodontic treatment**Circle one of the answers, write numbers or text

| 27. | What do you call a doctor who specializes in treatment of periodontal tissue diseases called (circle)? | General practice dentist | Orthopedist | | Oral and maxillofacial surgeon  Periodontologist* | Endodontist  Orthodontist | | I do not know |
| --- | --- | --- | --- | --- | --- | --- | --- | --- |
| 28. | What is the primary cause of periodontal diseases? | Poor diet | Genetic and heredity | | Dental plaque and calculus* | Dental trauma | | I do not know |
| 29. | How often is it recommended to get for professional oral hygiene? | Once or more per year | Once per year | | Twice per year* |  | | I do not know |
| 30. | Do systemic diseases have influence on periodontal tissues? | Yes, it does* | No, it does not | |  | | | I do not know |
| 31. | What it is the effect of smoking on periodontal tissues? | It has negative effect* | It does not effect | | It has positive effect |  | | I do not know |
| 32. | What symptoms show the start of gingival inflammation (gingivitis)? | Gum bleeding, ache swell* | New spaces between teeth | | Increase of teeth mobility |  | | I do not know |
| 33. | Is it possible, to lose a tooth, because of progressive periodontal disease which has not been treated? | Yes* | No | | |  | | I do not know |
| 34. | Does pregnancy have any influence on periodontal diseases? | Yes* | No | |  |  | | I do not know |
| 35. | Which methods are used for the treatment of periodontal diseases? | Caries treatment, esthetic restoration | Implantation, removal of cysts | | Professional oral hygiene, deep gingival cleaning* | Tooth root canals treatment and restoration | | I do not know |
| 36. | Did you know that it is possible to get orthodontic treatment while having periodontal diseases? | Yes* | No | |  | |  | I do not know |
|  |  | **IF “YES” THEN FILL BELOW** | | | | | |  |
| 37. | Do you wish to undergo orthodontic treatment? | Yes  For what reason (underline)?  To improve esthetic appearance and functional aspects, to maintain your own teeth  other................................................................ | | | No  (If no, then why?)  ………………………………………  ……………………………….…….. | | | I do not know |
| 38. | Would you agree to pay for orthodontic treatment (underline)? | Yes ≤ 2000€ | | Yes ≤3000 € | Yes ≤5000 € | No | |  |
| 39. | Which appliances would you agree to have as a way of treatment (multiple answers)? | Braces | | Clear aligners | I do not want to get orthodontic treatment. I wish to extract teeth and to get implants. | | |  |
| 40. | What aspects make you feel concerned about orthodontic treatment? | The look of appliances | | Treatment time | Regular visits | Price | | Other ……………………….. |
| 41. | Which length of orthodontic treatment suits you the best? | 6 months | | 1 year | 2 years | 3–4 years | | Does not matter |
| 42. | Would you agree to improve your oral hygiene during the orthodontic treatment? | Yes | No | |  | | | |
| 43. | What kind of information about orthodontic treatment would you need (write)? | ………………………………………………………………………………………  ………………………………………………………………………………………  ……………………………………………………………………………………… | | | | | | |
| 44. | Can we contact you about the orthodontic treatment? | Yes  If “Yes” mention your contact information  below:  E- mail …………………….  Phone:………………………….. | | | | | No | |

* Correct answer to questions (no. 27–35) to test knowledge in periodontal disease etiology (the symbol * is not shown to respondents).
